# Automatic Classification of Simulated Breast Tomosynthesis Whole Images for the Presence of Microcalcification Clusters Using Deep CNNs

**DOI:** 10.3390/jimaging8090231

**Published:** 2022-08-29

**Authors:** Ana M. Mota, Matthew J. Clarkson, Pedro Almeida, Nuno Matela

**Affiliations:** 1 Instituto de Biofísica e Engenharia Biomédica, Faculdade de Ciências, Universidade de Lisboa, 1749-016 Lisboa, Portugal; 2 Department of Medical Physics and Biomedical Engineering and the Centre for Medical Image Computing, University College London, London WC1E 6BT, UK

**Keywords:** digital breast tomosynthesis, microcalcifications, deep-learning, convolutional neural network, virtual clinical trial

## Abstract

Microcalcification clusters (MCs) are among the most important biomarkers for breast cancer, especially in cases of nonpalpable lesions. The vast majority of deep learning studies on digital breast tomosynthesis (DBT) are focused on detecting and classifying lesions, especially soft-tissue lesions, in small regions of interest previously selected. Only about 25% of the studies are specific to MCs, and all of them are based on the classification of small preselected regions. Classifying the whole image according to the presence or absence of MCs is a difficult task due to the size of MCs and all the information present in an entire image. A completely automatic and direct classification, which receives the entire image, without prior identification of any regions, is crucial for the usefulness of these techniques in a real clinical and screening environment. The main purpose of this work is to implement and evaluate the performance of convolutional neural networks (CNNs) regarding an automatic classification of a complete DBT image for the presence or absence of MCs (without any prior identification of regions). In this work, four popular deep CNNs are trained and compared with a new architecture proposed by us. The main task of these trainings was the classification of DBT cases by absence or presence of MCs. A public database of realistic simulated data was used, and the whole DBT image was taken into account as input. DBT data were considered without and with preprocessing (to study the impact of noise reduction and contrast enhancement methods on the evaluation of MCs with CNNs). The area under the receiver operating characteristic curve (AUC) was used to evaluate the performance. Very promising results were achieved with a maximum AUC of 94.19% for the GoogLeNet. The second-best AUC value was obtained with a new implemented network, CNN-a, with 91.17%. This CNN had the particularity of also being the fastest, thus becoming a very interesting model to be considered in other studies. With this work, encouraging outcomes were achieved in this regard, obtaining similar results to other studies for the detection of larger lesions such as masses. Moreover, given the difficulty of visualizing the MCs, which are often spread over several slices, this work may have an important impact on the clinical analysis of DBT images.

## 1. Introduction

Breast cancer is the most commonly diagnosed type of cancer worldwide [1]. Over the last three decades, mortality rates for breast cancer have dropped from their peak by 41%, likely reflecting advancements in treatment and earlier detection through increased screening programs [2]. However, in women, this disease is still the leading cause of cancer death [1].

Breast screening is crucial in identifying breast cancer at an early stage, when it can be better located and treated, thus reducing the breast cancer mortality. It is estimated that women who chose to participate in an organized breast cancer screening programs have 60% lower risk of dying from breast cancer within 10 years after diagnosis [3]. Until recently, these screenings and breast cancer detection in general were mainly performed using digital mammography (DM). However, as a result of its 2D nature, DM presents two major limitations: low sensitivity in dense breasts with pathology and low specificity due to normal tissue superposition [4].

The use of digital breast tomosynthesis (DBT) has been confirming the potential of DBT to address these limitations. Initially, DBT was studied and approved in conjunction with DM, demonstrating an increase in breast cancer detection rates and a significant reduction in recall rates [4,5,6,7,8,9], particularly with dense breasts [6]. Currently, by including synthetic mammography (SM) generated from DBT data, DBT alone is approved as a stand-alone modality to replace DM [10,11,12,13,14,15].

One major drawback with DBT is its increase in interpretation time when compared to DM [16,17]. Computer-aided detection (CAD) systems with DBT were implemented and evaluated in an attempt to shorten the reading time while maintaining the radiologist performance. In fact, some results are very encouraging with reading time reductions between 14% and 29.2% without loss of diagnostic performance [18,19,20].

On the other hand, there are mixed observations with respect to DBT technology for the detection of microcalcification clusters (MCs). Some studies have revealed inferior image quality for visibility of MCs with DBT [21,22,23] while others have not [24,25,26]. As MCs are among the most important biomarkers for breast cancer [27,28], especially in cases of nonpalpable lesions, another CAD approach that has been extensively studied with DBT is the use of these conventional CAD systems to assist in the correct detection of MCs [29,30,31,32,33,34,35,36,37]. However, despite the efforts and improvements already achieved (such as decreasing the false negative rate), due to the high false positive rates and low specificity, these CAD systems have not reached a level of performance that can be translated into a true improvement in the real screening of breast cancer [38,39,40,41].

In recent years, the increase in computational power has allowed the development of deep learning artificial intelligence (AI) algorithms composed of multilayered convolutional neural networks (CNNs). These AI systems have emerged as a potential solution in the field of automated breast cancer detection in DM and DBT [41]. In fact, recently, there have been several published large-scale studies where the aim was to analyze the performance of AI systems alone, as well as the performance of breast radiologists with and without AI [20,42,43,44,45,46,47,48,49]. The AI systems under evaluation achieved a comparable or even improved cancer detection accuracy when compared with the human experts. With these promising results and the need for an automatic detection system for lesions in DBT and in screening, much research has been carried out in this regard. A brief summary of these studies is presented in Table 1.

The vast majority of these studies focused on detecting and classifying soft-tissue lesions, such as masses [51,52,53,54,55,56,57,64,65]. In addition to the fact that these are important lesions for the characterization of breast cancer, in this type of lesion, it is possible to greatly reduce the data input size through interpolation, without losing the spatial resolution required to observe the lesion (the same does not occur with MCs). In this way, faster transfer learning solutions, useful when there is a lack of available training data (as in the case of DBT), can be used with very positive results [53,54,55,56,64,65]. Even in cases where only regions of interest (ROIs) and not full images are selected, such resizing is usually carried out. Furthermore, the vast majority of the works use ROIs or patches where objectively there is or is not a lesion [55,57,60,61,66,67,69,70], instead of using the whole image or volume. The use of the whole image or volume is important to contextualize the lesions but also to make the classification a useful and quick tool in screening, where an image/volume should ideally give some type of direct outcome.

One of the biggest challenges involving DBT in AI is the lack of a large, properly labeled public database. All studies mentioned in the Table 1, except one [71], used private databases, making generalization and a fair comparison between different studies impractical [72]. Recently, two publicly accessible annotated DBT datasets that will facilitate the evaluation and validation of AI algorithms were released. Buda et al. made publicly available a large-scale dataset of DBT data. It contains 5610 studies from 5060 patients: 5129 normal cases (no abnormal findings), 280 cases where additional imaging was needed but no biopsy was performed, 112 benign biopsied cases, and 89 cases with proven cancer. This dataset includes masses and architectural distortions and was used to train and test a single-phase deep learning detection model that reached a baseline sensitivity of 65% at two false positives per DBT volume [73]. El-Shazli et al. used this database to propose a computer-aided multiclass diagnosis system for classifying DBT slices as benign, malignant, or normal considering masses and architectural distortions [71]. The other public dataset resulted from the advancement of in silico tools. The Virtual Imaging Clinical Trial for Regulatory Evaluation (VICTRE) project was created for the evaluation of the imaging performance of DBT as a replacement to DM for breast cancer screening. In VICTRE, the whole imaging chain was simulated with state-of-the-art tools, and a total of 2986 virtual realistic patients were generated and imaged with both modalities. The positive cohort (that comprises malignant spiculated masses and MCs) included 1944 and 1042 virtual patients with and without lesions, respectively [74].

In this paper, fully automatic methods based on deep learning were studied for classifying DBT data. The aim is to input a whole DBT image and have a direct answer about the absence or presence of MCs, without the need for prior identification of lesions in specific regions and, thus, completely automate the process of DBT classification. Four existing popular networks were considered and compared with a new network proposed by us for this purpose. In order to study the impact of some preprocessing methods in increasing the visibility of MCs, the input data were considered with and without preprocessing. The VICTRE public database was used. To the best of our knowledge, this is the first study of automatic classification specifically dedicated to the presence or absence of MCs in whole DBT images.

## 2. Materials and Methods

### 2.1. Database

This study was centered on the database created for the VICTRE trial [74]. Synthetic images of virtual patients were obtained using an in silico version of the Siemens Mammomat Inspiration DBT system using Monte Carlo X-ray simulations. These data are available to the public in the Cancer Imaging Archives [75]. Physical compression of left breasts was considered in the craniocaudal (CC) orientation. In this database, the cases are divided into the absence and presence of lesions, as well as according to the density of the breast (fatty, scattered, heterogeneous, and dense). The absent cases have no findings, and each case with lesions present contains four spiked masses with a 5 mm nominal diameter and mass density 2% higher than normal glandular tissue, and four MCs consisting of five calcified lesions modelled as 195, 179, and 171 μm of solid calcium oxalate. In this study, we included cases without (“absent”) and with MCs (“present MCs”).

Table 2 presents a detailed summary of the dataset selected for this work. The reconstructed cases had different dimension in *x*, *y*, and *z*, depending on breast density: 1624×1324×62, 1421×1024×57, 1148×753×47, and 1130×477×38 for fatty, scattered, heterogeneous, and dense breasts, respectively, with a voxel size of $0.085\times 0.085\times 1{\;\mathrm{mm}}^{3}$. For the absent category, five slices proportionally spaced between the first and the last slice were selected for each case (for example, as fatty cases have 62 slices: slices 1, 17, 33, 49, and 62 were selected; as dense cases have 38 slices: slices 1, 11, 21, 31, and 38 were chosen). On the other hand, for the presentMCs class, slices containing the center of the cluster were selected for each case (in some cases, two clusters had their center on the same slice). Numerically, we adopted the usual distribution of breast density in the population: 10% fatty, 40% scattered, 40% heterogeneous, and 10% dense, with an approximate balance between cases without and with lesions.

### 2.2. Data PreProcessing

In the VICTRE database, the reconstructed data have signal contamination outside the breast region, i.e., in the background (BG). This information is worthless for training the networks and, when present, slows down the process, as pixels without any useful information end up contributing to the mathematical operations involved. In this way, through binarization and region-growing operations, binary masks that keep information belonging to the breast and make everything else zeros were created (“BG suppression”). This step was applied to the original data and after all the other types of processing.

**Figure 1:**
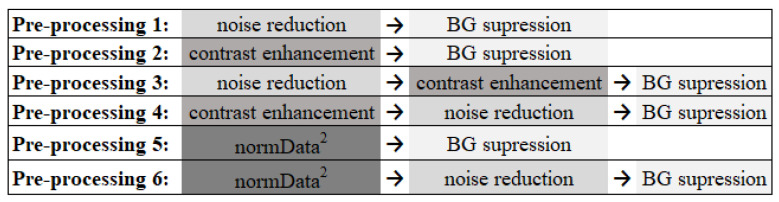
The six preprocessing methodologies implemented in order to reduce noise and amplify the visibility of the MCs (BG: background, normData: data normalized between 0 and 1).

The very-low-dose projections acquired within a limited angular range in a DBT examination result in low statistics (high noise level) in the reconstructed images and data insufficiency. For this reason, image denoising methods are very important in order to improve the image quality of DBT data. Total variation (TV) minimization algorithms have attracted considerable attention in the field because of their ability to smooth images while preserving the edges. Studies applying TV minimization to DBT data have shown excellent results so far [76,77,78,79,80]. This methodology was applied during the preprocessing step. Minimization of TV greatly improves the contrast-to-noise ratio by reducing the noise. In this way, in order to also increase the contrast, two other techniques were studied. The contrast-limited adaptive histogram equalization (CLAHE) technique was implemented to increase the contrast of all breast structures in general, and a simpler operation was applied to increase the contrast of structures with greater intensity, such as MCs, in particular. Since we wanted to study whether image noise reduction or contrast has any impact on CNN training, some combinations of these methods were made, resulting in six different preprocessing approaches (Figure 1), as described below.

**PreProcessing 1**: As DBT data are composed of a high level of noise resulting from the acquisition of low-dose projections, the application of a noise reduction filter was analyzed. This filter consists of minimizing the TV of the data, allowing the noise to be significantly reduced while preserving the edges and lesion resolution (which is a very important factor when the structures under analysis are small MCs). TV is a measure of pixel intensity variation in an image and increases significantly in the presence of noise. In each preprocessing that included this filter, several Lagrange multipliers were tested to study which allowed the minimum TV value [78], and 14 was the chosen value for the application of the filter in all cases.

**PreProcessing 2**: The CLAHE technique [81] was implemented using the MATLAB R2020a function adapthisteq [82] to enhance the contrast of the images and the MCs. With this technique, the contrast in homogeneous areas is limited to avoid the amplification of noise. The contrast transformation function is calculated in small regions of the image individually, rather than in the whole image, and neighboring regions are then combined through bilinear interpolation to eliminate artificially induced boundaries. The contrast enhancement limit was 0.01, and a uniform distribution of the histogram was used with a distribution parameter of 0.4.

**PreProcessing3 and 4**: The techniques described for preprocessing 1 and 2 were combined and used together by varying the order in which each one was applied. These steps (3 and 4) were also included since techniques 1 and 2 could complement each other and, through preliminary studies, it was possible to conclude that their order of implementation showed differences in the appearance of the final image. In preprocessing 3, the TV minimization filter for noise reduction was first applied, followed by the contrast enhancement technique. For preprocessing 4, the application was in the opposite order, with contrast enhancement technique first and then noise reduction.

**PreProcessing 5**: The data intensity was first normalized between 0 and 1 and then squared to attenuate the lower values, highlighting the higher ones belonging to the MCs. With this filter, our aim was to specifically increase the contrast of regions of higher intensities.

**PreProcessing 6**: The method applied in preprocessing 5 was followed by the TV minimization filter, as described in preprocessing 1.

In order to homogenize the data, as well as to find a balance between training time/memory and the necessary spatial resolution for the visibility and conspicuity of MCs, all data were resized in *x* and *y* to 512×512. No adjustments were made in the *z*-direction since training was performed slice-by-slice. The images were converted into TIFF slices of 8 bits, and input data were normalized using the zero center method.

### 2.3. CNNs

Since it was crucial to maintain image spatial resolution under certain limits to allow the detection of the small MCs, it was not possible to reduce the image dimension to values such as 224×224 or 227×227, which are the most used in pretrained networks for transfer learning. Our approach was then to train from scratch four architectures that already exist: AlexNet [83], GoogLeNet [84], ResNet18 [85], and SqueezeNet [86]. The choice of these popular networks was based on the comparison of each model’s speed and accuracy [87].

**Figure 2:**
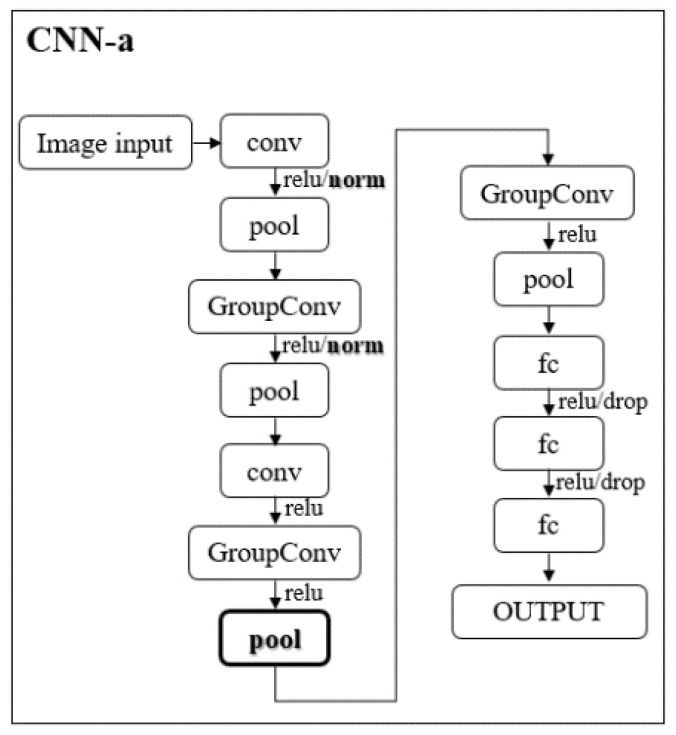
Illustration of CNN-a that resulted from the modifications made (bold) to the AlexNet architecture. Conv and GroupConv: convolutional and grouped convolutional layers, respectively; pool: max pooling layers; fc: fully connected layer; relu: rectified linear unit layer; norm: batch normalization layer; drop: dropout layer.

In addition, to alleviate some computational effort, one faster and lighter new architecture, based on AlexNet, is proposed by us: CNN-a (Figure 2).

In CNN-a, the channel-wise local response normalization layers were replaced by batch normalization layers (“norm”) and a new max pooling layer with a stride of 2, padding of 0, and size of 3 × 3 was added between the two grouped convolutional layers. These modifications were the result of several empirical trial-and-error studies conducted by us during the experiment.

### 2.4. Methodology Pipeline

**Figure 3:**
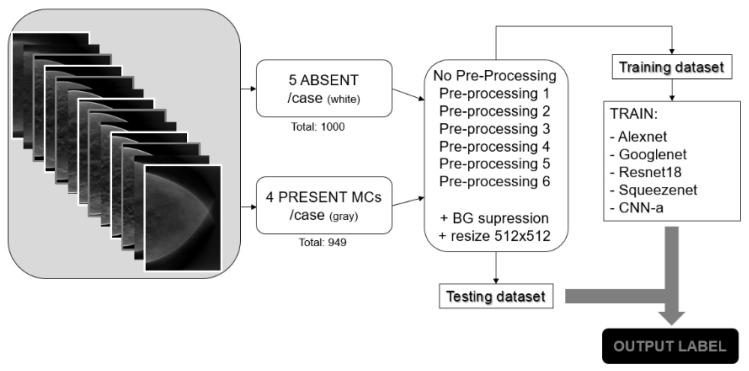
Summary of the methodological pipeline followed in this work.

Figure 3 shows the pipeline followed in this work. Absent and presentMCs data samples were selected, and the described preprocessing techniques were applied. The training dataset was used to train the CNNs from scratch, and the testing dataset was used after training to evaluate the performance of the trained CNNs.

### 2.5. Training Options

The *k*-fold technique was used as the cross-validation method to estimate the generalization error of the learning process. The dataset used was divided into *k* = 3 subsets, i.e., each network was trained and tested three times with different datasets, always according to the proportion of two-thirds of the cases for training and one-third for testing. Since the split was performed at the patient level, all the images of the same patient were in either the training set or the test set. Training data augmentation was used through random reflection in the left–right direction (to simulate the inclusion of examples of right breasts) and data rotation between ±20°.

The CNNs were trained using the stochastic gradient descent optimizer with momentum 0.9 to minimize the cross-entropy loss for classification. The maximum number of epochs was 200 with a mini-batch size of 32 and a learning rate of $1\times 10^{-3}$. In addition to the threefold cross-validation, an L2 regularization term of $5\times 10^{-3}$ was introduced in the loss function to prevent overfitting.

### 2.6. Evaluation Metrics

Classification problems usually involve distinguishing between two classes. In the case of medical imaging, this distinction is usually made between the absence or presence of abnormalities or between benign/malignant lesions. In our work, the objective was to distinguish between the absence or presence of MCs. Sensitivity, specificity, accuracy, and area under the receiver operating characteristic (ROC) curve (AUC) were considered to evaluate the performance. The analysis of only the first three metrics can be limitative because they depend on the defined threshold to accept a case as presentMCs or absent. In this way, we used the AUC (positive class: presentMCs) as a summary tool that contains the space of all these possible thresholds.

Differences in the performance of each classifier were tested using a statistical *t*-test. A two-tailed *p*-value < 0.05 was considered to indicate a significant difference.

## 3. Results

### 3.1. Data Preprocessing

**Figure 4:**
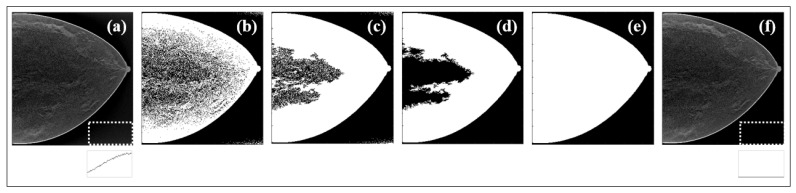
(**a**) Data with contaminated BG; (**b**) first binary image; (**c**) filled binary image; (**d**) largest object extracted from binary image; (**e**) result from region growing; (**f**) final image with BG corrected after binary mask from (**e**) applied to (**a**).

All the steps involved in the BG suppression are presented through an example case in Figure 4. The original data were first binarized (Figure 4b) by thresholding, the holes in the image were filled (Figure 4c), the largest resultant object was selected (Figure 4d), and the complete binary mask was achieved by performing region growing in (Figure 4e). The profile traced for the white ROI (lower right corner of (a) and (f)) shows the cleaning effect.

**Figure 5:**
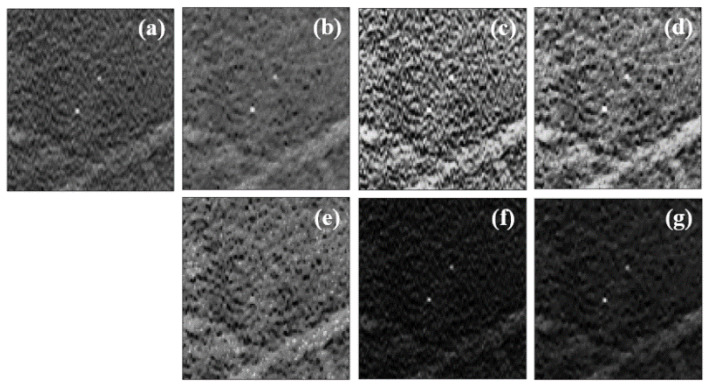
(**a**) Original data without preprocessing; (**b**) preprocessing 1 (minimization of TV); (**c**) preprocessing 2 (CLAHE); (**d**) preprocessing 3 (minTV + CLAHE); (**e**) preprocessing 4 (CLAHE + minTV); (**f**) preprocessing 5 (dataNorm2); (**g**) preprocessing 6 (dataNorm2 + minTV).

This methodology was included in all preprocessing approaches, as mentioned in Section 2.2. Zooming in on one MC (Figure 5), we can see the different results achieved in this type of lesions with each preprocessing method.

### 3.2. Performance Analysis

Our research was guided by the AUC results obtained for the different architectures and preprocessing methods. As mentioned above, the training and testing were repeated three times (threefold cross-validation) using three distinct datasets. The averaged performances and standard deviation values found over the three folds are shown in Table 3.

In Table 4 presents the *p*-values calculated to study the measurable statistical differences between the best mean AUCs obtained in Table 3.

**Figure 6:**
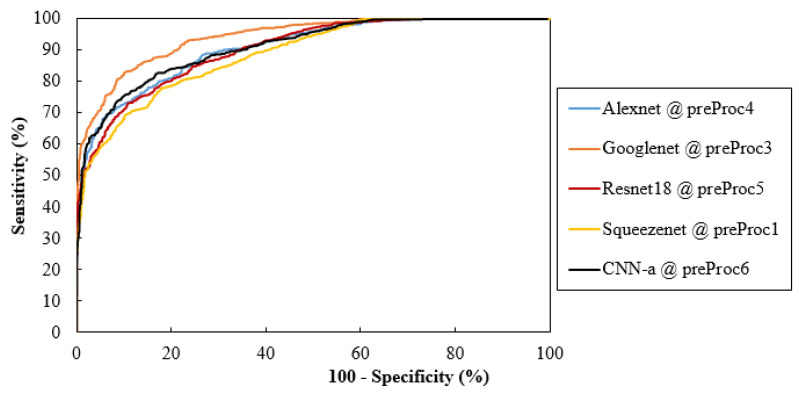
Comparisons of ROC curves for the CNNs and training data with the best AUC values; preProc—preProcessing.

**Figure 7:**
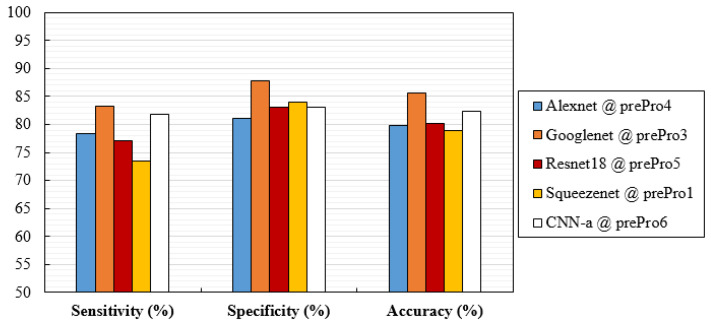
Values of sensitivity, specificity, and accuracy obtained with the architectures trained with preprocessed data that achieved the best mean AUC.

Considering only the best results obtained for averaged AUC, Figure 6 shows the ROC curves of the CNN network trained with the respective data. These curves were obtained by averaging between the ROC curves of each fold. Additionally, Figure 7 analyzes the values of the respective sensitivities, specificities, and accuracy in detecting the cases with MCs.

### 3.3. Influence of Breast Density on Classification

**Figure 8:**
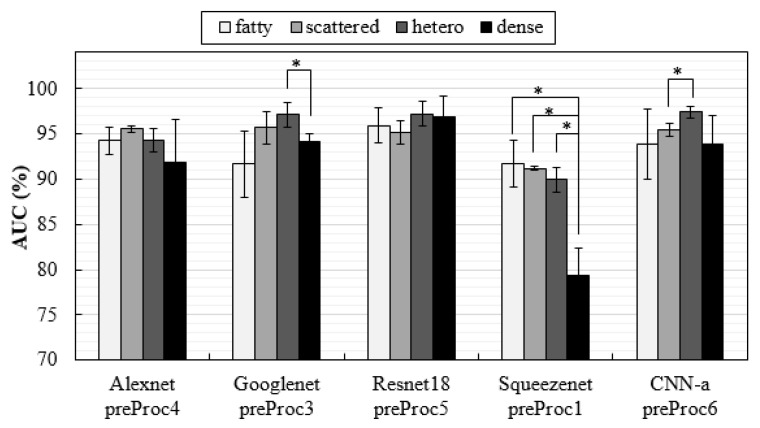
AUC values obtained with test datasets composed by the four different breast densities separately (* p<0.05 indicates a significant difference between groups).

Breast density interferes with the detection of lesions [88]. In this way, it was important to explore the influence of density on the specific detection of MCs with these CNNs trained by these datasets. For this purpose, the training dataset were not changed, i.e., the CNNs were trained including all breast densities, but they were tested separately with specific datasets for each breast density. The results, in form of AUC values, are shown in Figure 8.

The training that provided the best performance (GoogLeNet @ preprocessing 3) required a training time of approximately 9 h for all three folds (using an NVIDIA Quadro P4000 GPU). On the other hand, the fastest training and second-best performance were obtained, simultaneously, for our CNN-a with data from preprocessing 6. Table 5 shows the training and inference times for all CNNs.

## 4. Discussion

In this work, the training from scratch of four popular CNNs and a new architecture proposed by us was investigated. Given the whole DBT image (and not only some specific ROIs) as input, the classification of cases by absence or presence of MCs was the main task of these trainings. Original data and data resulting from preprocessing methods (to increase MCs visibility) were considered. The DBT dataset used for training and testing are from the public database available at The Cancer Imaging Archive website [75].

In order to avoid useless complex mathematical operations, all the information outside the breast region was eliminated. In four steps, an automatic methodology that creates a binary image where only the information inside the breast is considered was implemented. The comparison between the contaminated data and the data with complete suppression of BG signal can be observed through the profiles of the yellow regions in Figure 4a and Figure 4f, respectively. This operation represented a difference of about 5% in training times, without performance losses, and it is usually applied in this type of CNN training.

Data preprocessing can be very useful when training CNNs from scratch to facilitate the detection and classification processes. In this work, both original data and data resulting from different preprocessing methods were considered as input. A comprehensive study of different methods to make the MCs more visible to the algorithms was carried out.

In original data, the MCs showed reasonable contrast to the naked eye (Figure 5a). This highlight can be compromised due to their size, the presence of noise, and other structures that can make them less visible. Both preprocessing 1 and preprocessing 2, had a great influence on MCs data. Preprocessing 1 smoothed the region around the MCs, preserving its edges (Figure 5b), while preprocessing 2 contributed to an increase in contrast between all structures, whether they were MCs or not (Figure 5c). We thought it might be interesting to combine a technique that is essentially for noise reduction (TV minimization) with a CLAHE technique; in this way, preprocessing 3 and preprocessing 4 corresponding to Figure 5d and Figure 5e, respectively, were implemented. While, visually, the MCs stand out from the surrounding noise in Figure 5d, in Figure 5e, where the contrast enhancement was applied first and the noise reduction latter, the MCs appear to fade. Additionally, for its simplicity, another method based on squared normalized data was also studied (preprocessing 5). This operation worked quite well when it comes to highlighting high-intensity structures (Figure 5f). The application of the TV minimization filter to these data (preprocessing 6) also resulted in a reduction in anatomical noise that allowed for greater differentiation of the MCs, as can be seen in Figure 5g.

**Figure 9:**
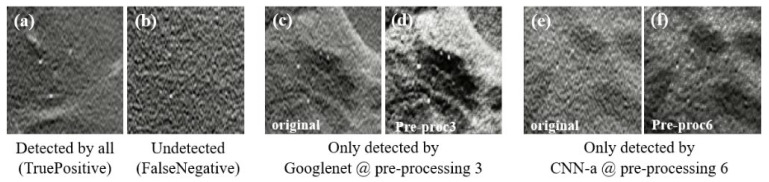
Some examples of MCs in the DBT data used. (**a**) True positive (case correctly classified as positive by all CNNs, even in the original image); (**b**) false negative (case incorrectly classified as negative by all CNNs, even when varying the preprocessing); (**c**) original case classified as negative and that was only detected by GoogLeNet when preprocessed with method 3 (**d**); (**e**) original case classified as negative that was only detected by CNN-a when preprocessed with method 6 (**f**).

This descriptive analysis is in line with the numerical results obtained for the trained CNNs. From Table 3, it can be seen that the results were affected not only by the type of input data, but also the CNN architecture itself. In fact, the best AUC value of each CNN was achieved with different input data. GoogLeNet showed the best AUC with data processed using method 3 (94.19%), CNN-a showed the best AUC with data processed using method 6 (91.17%), AlexNet showed the best AUC with data processed using method 4 (90.82%), ResNet18 showed the best AUC with data processed using method 5 (90.44%), and SqueezeNet showed the best AUC with data processed using method 1 (88.78%). CNNs trained with original data did not generate a maximum AUC. However, all the AUC values were higher than 86%, showing that, even without any preprocessing, this could be an option. As shown in Figure 9a, for cases where the MCs were in a region with less noise and were more evident, all the CNNs achieved a correct classification in the original data. On the other hand, despite the efforts to reduce noise and increase contrast, some cases such as the one in Figure 9b were incorrectly classified as negative by all CNNs, even when varying the pre-processing. Although preprocessing 2 did not contribute to a maximum either, it resulted in the third-best AUC for GoogLeNet. From Table 3, it is also possible to conclude that GoogLeNet was the most sensitive CNN to data contrast since its best results of AUC were obtained with methods where the contrast enhancement operation was performed. In the example of a case where MCs were in a region with other structures also of greater contrast (Figure 9c,d), GoogLeNet took advantage of preprocessing 3 and was the only CNN to correctly classify this case. As a matter of fact, the GoogLeNet trained with data processed using method 3 presented significantly higher values in the detection of cases with MCs (*p*-value < 0.05, Table 4). This superiority is quite visible in the isolated ROC curve in Figure 6. The second-best performance corresponded to CNN-a trained with data from preprocessing 6, with this superiority significant in relation to ResNet18 and SqueezeNet (Table 4). In Figure 9e there is a case of a MCs that were masked and only detected by CNN-a after preprocessing 6 (Figure 9f). Thus, in agreement with the results in Table 3, we can assume that it is the combination of both factors (data type and CNN) that determines the result of a correct classification.

The variations and differences in AUC values obtained for each situation were, in general, in agreement with the specificity, sensitivity, and accuracy values obtained in Figure 7. Although specificity values were higher than sensitivity in most cases, these differences were not significant (*p*-value > 0.05 in all cases). As for accuracy, GoogLeNet and CNN-a presented the best values of 85.68% and 82.45%, respectively.

In the VICTRE database, it is possible to separate the cases by breast density, and a study was published where a model observer was trained separately for detecting lesions in each of the four breast density types and then tested on the same density type to obtain the individual AUC for each density [89]. As a conclusion of this study, Zeng et al. believed it would be appropriate to train the model observer with mixed breast density images. This was exactly what we did with the deep learning architectures proposed in this work. However, in order to understand whether the presented methodologies were influenced or not by breast density, the same CNNs were tested separately for classifying the DBT data about the presence of MCs in each of the four breast density types (fatty, scattered, heterogeneous, and dense), and the results were analyzed in terms of AUC. As seen in Figure 8, only SqueezeNet was especially sensitive to density, showing significant differences in detection among the three density types. The correct classification of cases with MCs in dense breasts with SqueezeNet was significantly lower compared to the other densities. In general, due to the lower anatomical background, fatty breasts allowed good classifications of cases with MCs. GoogLeNet was the exception, with fatty breasts corresponding to the lowest AUC value (*p* > 0.05).

Training and inference times of Table 5 are purely indicative as they vary depending on the computation power available. However, in relative terms, the already existent networks (GoogLeNet, ResNet18, SqueezeNet, and AlexNet) led with the four longest times. On the other hand, although the CNN with the best AUC (GoogLeNet) showed the longest time, the second best (CNN-a) was the faster network. As inference time is the key when the models are used in clinic, it should be noted that, with CNN-a, it was possible to classify an image never seen by the model before about three times faster than with GoogLeNet. From our point of view, this fact makes this architecture adapted from AlexNet very interesting for future studies that involve more complex and longer trainings, such as object detection with state-of-the-art faster region-based CNNs. One of the most determining factors in the training/testing time of these CNNs is the feature extraction network that is used as the basis. Thus, a faster model such as CNN-a, which presents good results regarding the classification of cases with MCs, should be an option to be studied in the future.

In two published studies (2D and 3D), where a prescreening stage generates possible MCs and the proposed CNNs differentiate between true MCs and false positives, AUC values of 93% [50] and 97.65% [68] were reported. Both studies used ROIs instead of the whole image/volume. Some regions do not have any lesions or relevant information, while others contain only the lesions. On the other hand, in a study where the main objective was to compare the detection of MCs in images reconstructed with two different reconstruction algorithms (EMPIRE and filtered back projection), small 3D patches were used as input, and the best result obtained in terms of partial AUC was 88.0% [58].

In another study, an ROI was selected for each lesion on a DBT key slice, features were extracted using a pretrained CNN and served as input to a support vector machine classifier trained in the task of predicting likelihood of malignancy [62]. The AUC result obtained in CC view for MCs detection was 82%. Other views were included, and, considering MLO (mediolateral oblique) in addition to CC view, AUC improved to 97%, showing the importance of having both views available.

Xiao et al. proposed an interesting ensemble CNN to classify benign and malignant MCs in DBT. This classification was made on smaller patches (300 × 300) containing only the MCs. The AUC and accuracy using a decision-level ensemble strategy were 0.8837 and 0.82, respectively [70].

The only work that took the whole image information into account used 2D synthetic mammographic images obtained from DBT exams to train a multi-view deep CNN to classify screening images into BI RADS classes (0: further evaluation is required due to a suspicious abnormality; 1: the mammogram is negative; 2: the mammogram is benign). The AUC values obtained were as follows: BI-RADS 0 vs. others, 91.2%; BI-RADS 1 vs. others, 90.5%; BIRADS 2 vs. others, 90.0% [66].

A direct comparison between literature values and those obtained in this work is not fair due to several reasons. The first is that different databases were used (those of the studies mentioned were all private databases). The second is that the training data have quite different characteristics due to different detection tasks. Some used only small parts of the data, and those which used the entire image did not refer to DBT slices but rather to synthetic mammograms obtained with DBT. Nevertheless, it is possible to confirm that the results obtained by our study (maximum value of AUC achieved: 94.19%) are quite competitive when compared to those available in the literature.

There were some limitations in this study. The first is that the available dataset is limited to the CC view and one manufacturer. The second is that only one type of lesion (MCs) was considered, and, within the available data, there may be some similarities between lesions. We tried to overcome this fact through data augmentation with reflection and rotation. The third is that, despite being very realistic, the data are simulated and, therefore, do not correspond to real patients. Lastly, since DBT is a 3D technique, the fact that we consider information in 2D slices can limit the advantage provided by the depth information. Furthermore, the true clinical value lies in the classification of a volume, because this is what radiologists do every day in clinical practice. We believe that this work is a starting point and can serve as a basis for the implementation of a 3D training with all volume and 3D architectures, considering real data volumes and not just some slices. In addition, it will also be important to diversify the lesions, including data obtained from other views (MLO), manufacturers, and reconstruction algorithms. As for the training of the CNNs themselves, other optimizers that have been producing good results (such as Adam optimizer), as well as different mini-batch sizes and learning rates, should be tested and evaluated.

## 5. Conclusions

Deep learning AI algorithms composed of multilayered CNNs have been growing over the past 5 years and have shown very promising results in supporting the detection of breast cancer. One of the great difficulties in training these algorithms is the lack of labeled DBT databases. Furthermore, all published studies refer to private databases, thus limiting the comparison and improvement of the studies carried out.

In this study, a public DBT dataset was used to train from scratch four popular CNNs and a new CNN model proposed by us. The main task of our algorithms was to classify a DBT case for the presence or absence of MCs, given the whole DBT image as input. In addition to the original data, six different preprocessing methodologies, the main purpose of which was to highlight MCs, were implemented to generate different input datasets.

Classifying the whole image according to the presence or absence of MCs is a difficult task due to the size of MCs and all the information present in an entire image. With this work, we were able to achieve encouraging outcomes in this regard, obtaining similar results to other studies for the detection of larger lesions such as masses. The classification of cases with/without MCs was greatly influenced by the type of input data, and our new model achieved the second-best performance in the shortest time, thus becoming a very interesting model to be considered in future studies.

## Figures and Tables

**Table 1:** Summary of deep learning DBT studies (ROI: region of interest, AUC: area under the curve, pAUC: partial AUC).

Ref.	Classification Task	ROI/Patch/Image	Model	Best Metric
[50]	True MCs vs. false positives	ROI (16 × 16)	Own	AUC: 0.93
[51]	Presence/absence of masses and architectural distortions	Patch (256 × 256)	Based on AlexNet	Accuracy: 0.8640
[52]	Presence/absence of masses	ROI (32 × 32 × 25)	Own	AUC: 0.847
[53]	True masses vs. false positives	ROI (128 × 128)	Own	AUC: 0.90
[54]	True masses vs. false positives	ROI (64 × 64)	Based on VGG16	AUC: 0.919
[55]	Positive (malignant, benign masses) vs. negative images	Image (224 × 224)	Based on AlexNet	AUC: 0.6632
[56]	Malignant vs. benign masses	ROI (128 × 128)	Based on AlexNet	AUC: 0.90
[57]	Malignant vs. benign masses	Image (256 × 256)	Own	AUC: 0.87
[58]	Presence/absence of MCs	Patch (29 × 29 × 9)	Based on [59]	pAUC: 0.880
[60]	Positive vs. negative volumes	Image (1024 × 1024)	Based on AlexNet, ResNet50, Xception	AUC: 0.854 (AlexNet)
[61]	Positive vs. negative volumes	Image (832 × 832)	Based on AlexNet, ResNet, DenseNet and SqueezeNet	AUC: 0.91 (DenseNet)
[62]	Benign vs. malignant lesions	ROI (224 × 224)	Based on VGG19	AUC (MCs): 0.97
[63]	Positive vs. negative patches	Patch (512 × 512)	Based on ResNet	AUC: 0.847
[64]	Malignant vs. benign vs. normal masses	ROI (256 × 256)	Based on VGG16	AUC: 0.917, 0.951, 0.993 (malignant, benign, normal)
[65]	Malignant vs. benign masses	ROI (224 × 224)	Based on DenseNet121	AUC: 0.8703
[66]	BIRADS 0 vs. BIRADS 1 vs. BIRADS 2	Image (2200 × 1600)	Based on ResNet50	AUC: 0.912 (BIRADS 0 vs. non-0)
[67]	Predict breast density	Image	Based on ResNet34	AUC: 0.952
[68]	True MCs vs. false positives	ROI (128 × 128)	Based on ResNet18	AUC: 0.9765
[69]	Malignant vs. benign vs. normal images	Image (150 × 150)	Own	AUC: 0.89
[70]	Malignant vs. benign MCs	Patch (224 × 224)	Ensemble CNN (2D ResNet34 and anisotropic 3D Resnet)	AUC: 0.8837
[71]	Malignant vs. benign vs. normal slices based on masses and architectural distortions	Image (input size of each CNN: 224 × 224, 227 × 227)	ResNet18, AlexNet, GoogLeNet, VGG16, MobileNetV2, DenseNet201, Mod_AlexNet	Accuracy: 0.9161 (Mod_AlexNet)

**Table 2:** Detailed summary of the VICTRE data selected for this study.

	Absent		Present MCs	
Density	Number of Cases	Number of Slices	Number of Cases	Number of Slices
Fatty	20	100	25	99
Scattered	80	400	100	386
Heterogeneous	80	400	100	371
Dense	20	100	25	93
**Total**		1000		949

**Table 3:** Performance results of CNNs trained with original data and with data resulting from the preprocessing methodologies, in terms of mean AUC.

	AUC (%): Mean ± SD				
	AlexNet	GoogLeNet	ResNet18	SqueezeNet	CNN-a
Original data	87.92 ± 2.01	90.14 ± 0.38	86.84 ± 2.62	87.43 ± 0.78	89.79 ± 1.23
Preprocessing 1	87.35 ± 1.63	88.38 ± 1.12	87.96 ± 0.96	88.78 ± 0.99	90.66 ± 0.15
Preprocessing 2	87.29 ± 0.78	93.02 ± 3.59	86.42 ± 3.26	86.84 ± 3.82	86.95 ± 0.97
Preprocessing 3	88.61 ± 0.43	94.19 ± 1.12	86.33 ± 1.46	82.15 ± 1.51	85.80 ± 1.73
Preprocessing 4	90.82 ± 1.29	94.15 ± 1.54	90.13 ± 0.32	86.33 ± 6.31	89.07 ± 1.62
Preprocessing 5	87.62 ± 0.35	88.65 ± 4.27	90.44 ± 0.41	85.18 ± 2.78	89.54 ± 2.63
Preprocessing 6	87.47 ± 1.13	89.76 ± 1.76	89.00 ± 1.33	84.09 ± 3.13	91.17 ± 0.07

**Table 4:** Levels of significance (*p*-values) obtained from the statistical analysis of the difference between the best mean AUCs found.

*p*-Value	GoogLeNet PreProc3	ResNet18 PreProc5	SqueezeNet PreProc1	CNN-a PreProc6
	(94.19 ± 1.12)	(90.44 ± 0.41)	(88.78 ± 0.99)	(91.17 ± 0.07)
AlexNet preProc4	**0.027**	0.654	0.095	0.662
(90.82 ± 1.29)	(AlexNet < GoogLeNet)			
GoogLeNet preProc3		**0.006**	**0.003**	**0.010**
(94.19 ± 1.12)		(GoogLeNet > ResNet18)	(GoogLeNet > SqueezeNet)	(GoogLeNet > CNN-a)
ResNet18 preProc5			0.055	**0.038**
(90.44 ± 0.41)				(ResNet18 < CNN-a)
SqueezeNet preProc1				**0.014**
(88.78 ± 0.99)				(SqueezeNet < CNN-a)

*p*-Values <0.05 (in bold) indicate a significant difference; preProc—preProcessing.

**Table 5:** Training times, in hours, needed for each CNN after threefold cross-validation and mean inference time (in seconds) needed to classify each image.

	Training Time (h)	Inference Time/Slice (s)
CNN-a	2.4	0.0057
AlexNet	4.1	0.0062
SqueezeNet	4.4	0.0083
ResNet18	7.8	0.0143
GoogLeNet	8.9	0.0158

## Data Availability

Data from VICTRE project was used. Available at https://wiki.cancerimagingarchive.net/display/Public/The+VICTRE+Trial%3A+Open-Source%2C+In-Silico+Clinical+Trial+For+Evaluating+Digital+Breast+Tomosynthesis.
